# Unraveling the Origin of Enhanced Activity of the Nb_2_O_5_/H_2_O_2_ System in the Elimination of Ciprofloxacin: Insights into the Role of Reactive Oxygen Species in Interface Processes

**DOI:** 10.1021/acsami.2c04743

**Published:** 2022-07-11

**Authors:** Lukasz Wolski, Kamila Sobańska, Malwina Muńko, Adrian Czerniak, Piotr Pietrzyk

**Affiliations:** † Faculty of Chemistry, Adam Mickiewicz University, Poznań, ul. Uniwersytetu Poznańskiego 8, 61-614 Poznań, Poland; ‡ Faculty of Chemistry, Jagiellonian University, ul. Gronostajowa 2, 30-387 Kraków, Poland; § Center for Advanced Technology, Adam Mickiewicz University, Poznań, ul. Uniwersytetu Poznańskiego 10, 61-614 Poznań, Poland

**Keywords:** niobium pentoxide, adsorption, antibiotics removal, advanced oxidation processes, photocatalytic regeneration

## Abstract

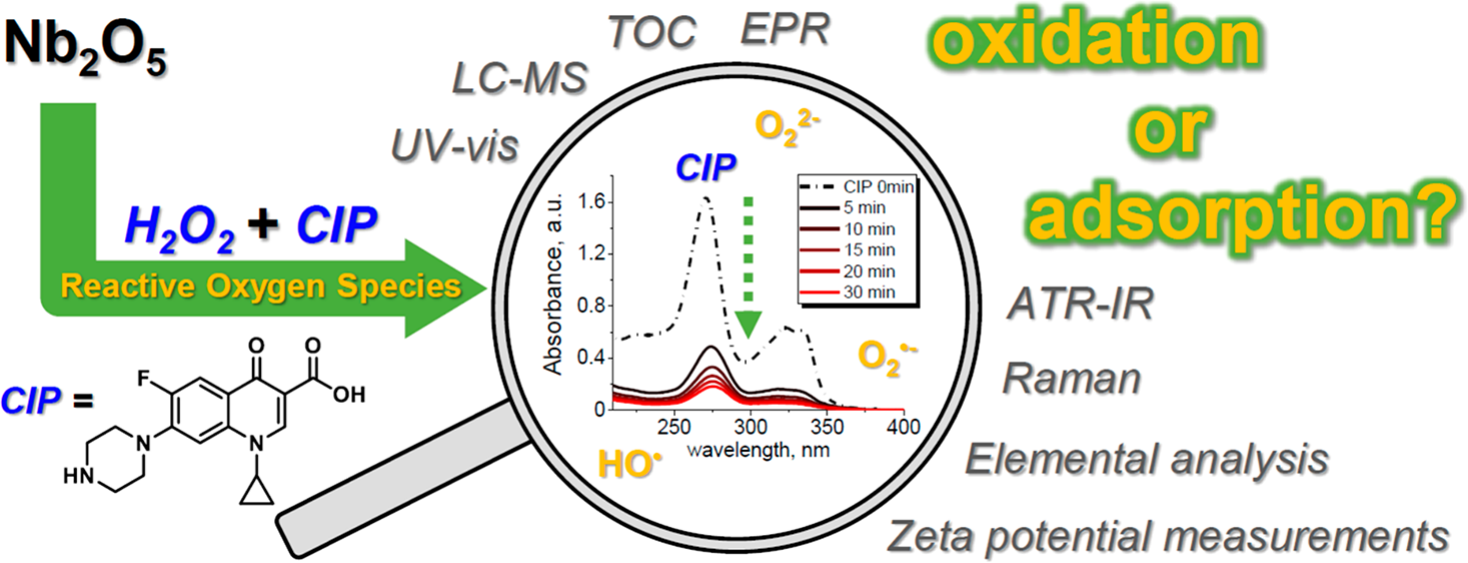

The overlooked role of reactive oxygen species (ROS), formed and stabilized on the surface of Nb_2_O_5_ after H_2_O_2_ treatment, was investigated in the adsorption and degradation of ciprofloxacin (CIP), a model antibiotic. The contribution of ROS to the elimination of CIP was assessed by using different niobia-based materials in which ROS were formed *in situ* or *ex situ*. The formation of ROS was confirmed by electron paramagnetic resonance (EPR) and Raman spectroscopy. The modification of the niobia surface charge by ROS was monitored with zeta potential measurements. The kinetics of CIP removal was followed by UV–vis spectroscopy, while identification of CIP degradation products and evaluation of their cytotoxicity were obtained with liquid chromatography–mass spectrometry (LC-MS) and microbiological studies, respectively. Superoxo and peroxo species were found to significantly improve the efficiency of CIP adsorption on Nb_2_O_5_ by modifying its surface charge. At the same time, it was found that improved removal of CIP in the dark and in the presence of H_2_O_2_ was mainly determined by the adsorption process. The enhanced adsorption was confirmed by infrared spectroscopy (IR), total organic carbon measurements (TOC), and elemental analysis. Efficient chemical degradation of adsorbed CIP was observed upon exposure of the Nb_2_O_5_/H_2_O_2_ system to UV light. Therefore, niobia is a promising inorganic adsorbent that exhibits enhanced sorption capacity toward CIP in the presence of H_2_O_2_ under dark conditions and can be easily regenerated in an environmentally benign way by irradiation with UV light.

## 1 Introduction

Antibiotics are common pharmaceuticals that are used not only as a medicine but also as growth promoters in agriculture, aquaculture, beekeeping, and livestock.^1^ Most of the antibiotic doses administered (from 40 to 90%, depending on the class of the drug) have been documented to be excreted from urine and feces as the parent substance (active form).^2^ The application of antibiotics on a worldwide scale and the difficulties in their elimination from industrial and municipal sewage result in the continuous discharge and accumulation of these chemicals in the environment. As a result, antibiotics can be detected not only in wastewater^3^ but also in natural ecosystems such as rivers and lakes.^1^ The continuous increase in the concentration of these pollutants in the environment poses a serious problem because it can contribute to the development of general resistance of bacteria to antibiotic treatment.^2^

These arguments show that the removal of excessive amounts of antibiotics from industrial and municipal sewage is a significant challenge. One of the most effective methods of their depletion from wastewater is the sorption process (immobilization of the antibiotic molecules on the surface of various adsorbents, e.g., activated carbons or chars,^4^ mineral clays,^5^ amorphous silica,^6^ or zeolites^7^). Its efficiency is strongly affected not only by the properties of the target pollutant and parameters of the wastewater (temperature, pH, presence of other pollutants, etc.) but also by the physicochemical characteristics of an adsorbent (e.g., surface area, surface acidity, isoelectric point, etc.).^1,8^ The most important drawbacks of sorption processes are associated with the need to regenerate the spent adsorbents. The regeneration process usually requires the use of other toxic chemicals, such as mineral acids, to desorb the immobilized antibiotic.^6,8^ Thus, the regeneration process may lead to the generation of additional corrosive waste, which must be neutralized anyway before being released into the environment.

Other highly efficient and promising methods for the elimination of antibiotics from wastewater are based on advanced oxidation processes (AOPs) that include photocatalytic degradation,^9,10^ Fenton and Fenton-like reactions,^11^ or ozonation.^12,13^ In all of these processes, antibiotic molecules are degraded (preferably mineralized) by highly oxidizing reactive oxygen species (ROS) formed *in situ* in reaction media. Recent reports in the literature have shown that AOPs can be used not only as an efficient approach to mineralize antibiotics^14^ but also as an environmentally benign method for the regeneration of spent adsorbents.^15−17^

Niobium pentoxide (Nb_2_O_5_) belongs to transition-metal oxides that are nontoxic, stable under acidic or alkaline conditions, and characterized by a large surface area and strong acidity.^18^ Nb_2_O_5_ exhibits the ability to activate H_2_O_2_ toward ROS formation combined with its stabilization on the surface.^19^ The type and amount of ROS formed in the Nb_2_O_5_–H_2_O_2_ system have been shown to be strongly affected by pH.^19^ The system is capable of producing hydroxyl radicals (^•^OH) and superoxide radical anions (O_2_^–•^) through the electroprotic mechanism. The first step of activation of H_2_O_2_ over Nb_2_O_5_ is the formation of HO_2_^–^ through the dissociation reaction: H_2_O_2(aq)_ + S–OH_(surf)_ → HO_2_^–^_(aq)_ + S–OH_2_^+^_(surf)_ (S stands for a surface site). As-formed HO_2_^–^ reacts in the liquid phase with H_2_O_2_ through the electroprotic reaction H_2_O_2(aq)_ + HO_2_^–^_(aq)_ → HO^•^_(aq)_ + O_2_^•–^_(aq)_ + H_2_O_(aq)_, leading to the generation of hydroxyl radicals and superoxide radical anions. The highest efficiency of this reaction is observed under acidic conditions (pH ∼ 3) and can be enhanced by two factors: (i) the presence of S–O^–^ species that are formed by dissociation of the surface hydroxyl groups (S–OH) at pH above the isoelectric point (IEP) of Nb_2_O_5_ and/or (ii) the adsorption and stabilization of superoxide radical anions on the surface of niobia. A similar mechanism has been proposed by us for amorphous ZrO_2_.^20^ At higher pH values, the main ROS formed are surface peroxide species (O_2_^2–^). Although numerous examples of applications to the degradation of dyes and pigments are available in the literature, reports on the application of this unique metal oxide in the elimination of pharmaceuticals are scarce.

The degradation of water-soluble pharmaceuticals in heterogeneous systems can be considered as a combination of two interdependent processes: physical adsorption (gauged by the extent of surface area, acidity, presence of specific surface groups, and surface charge of a solid) and chemical reactions driven by ROS, which are generated, for example, during interaction of H_2_O_2_ with the surface. As a result, such a surface must ensure the specific conditions necessary for the mutual and effective performance of both processes. Among ROS, hydroxyl radicals^21−23^ and superoxide anion radicals^24^ show high activity in complete oxidation processes, while in the case of selective oxidation processes peroxo surface forms are very often indicated.^25−28^ However, in addition to their chemical reactivity, charged ROS may influence the surface properties of catalysts^28^ and thus their ability to adsorb organic compounds. This, in turn, may have a significant impact on the efficiency of the degradation process because the adsorption of a pollutant is usually not only the first stage of its degradation but also constitutes a rate-limiting step of the entire process. Therefore, both processes, adsorption and oxidation, are linked by charged ROS. However, reports investigating the impact of ROS adsorption on the activity in environmental catalysis are lacking in the literature. The effect of surface modification (e.g., change of surface charge and ionic exchange) by adsorbed ROS is completely ignored, and the difference between the efficiency of removal of a toxicant in the presence or absence of H_2_O_2_ is assigned only to the degradation of organic compounds due to ROS oxidation. This approach may be wrong because it does not take into account the change of surface properties upon ROS adsorption, which can recursively influence both the chemical reactions and the adsorption process of an antibiotic.

Therefore, the main objective of this study was to investigate the specific role of reactive oxygen species, formed on Nb_2_O_5_ upon treatment with H_2_O_2_ in the dark, in the removal of ciprofloxacin (CIP) as a model antibiotic pollutant. Of particular interest was the evaluation of the contribution of adsorption and oxidative degradation to the removal of the antibiotic from wastewater under various conditions: (i) in the absence of hydrogen peroxide, (ii) in the presence of a small amount of H_2_O_2_ in the reaction medium (ROS formed *in situ* on Nb_2_O_5_), and (iii) with the use of a niobia catalyst that had previously been treated with concentrated H_2_O_2_ (ROS formed *ex situ* on the surface of Nb_2_O_5_) without hydrogen peroxide in the reaction medium. Furthermore, this study covers the identification of CIP degradation products formed in the dark and in the presence of H_2_O_2_ as well as after exposure of the spent niobia to UV irradiation. The latter was proposed as a simple and environmentally benign method for the regeneration of the spent niobia samples.

## 2 Experimental Section

### 2.1 Chemicals and Reagents

Reagents and chemicals used in the study were the following: ciprofloxacin (CIP, Sigma-Aldrich, purity ≥98%), nitric acid (HNO_3_, Chempur, 65%), sodium hydroxide (POCH, reagent grade), hydrogen peroxide (Sigma-Aldrich, 25–35% w/w in H_2_O, for ultratrace analysis), methanol (Merck KGaA, LC-MS grade), formic acid (Chem-Lab NV, 98–100%), 5,5-dimethyl-1-pyrroline *N*-oxide (DMPO, Sigma-Aldrich, ≥98%), and hydrochloric acid (HCl, POCH, 0.1 mol/L). All reagents were used without further purification. Deionized water was used throughout the experiments.

### 2.2 Preparation of Niobia Catalyst

In all experiments, amorphous niobium pentoxide (Nb_2_O_5_) supplied by Companhia Brasileira de Metallurgia e Mineração (CBMM) was used as the model catalyst. The BET surface area, the pore size distribution, and the low-temperature N_2_ adsorption–desorption isotherm recorded for this commercial catalyst are provided in the Supporting Information (Figure S1). The obtained surface area is equal to 155 m^2^/g. For the preparation of the niobia sample treated with concentrated hydrogen peroxide (sample with ROS formed *ex situ* on the surface of Nb_2_O_5_), 100 mg of powdered niobium pentoxide was spread inside a glass bottle in the form of a thin layer. Next, 500 μL of hydrogen peroxide (25–35% w/w in H_2_O) was added, and the bottle was placed in a dryer. After 18 h of drying at 60 °C, the yellow dry powder was obtained and used in catalytic tests. The prepared catalyst was labeled as Nb_2_O_5_/H_2_O_2_.

### 2.3 Characterization of Catalysts

To determine the surface charge of the niobia samples, measurements of the zeta potential as a function of the pH of the aqueous suspensions were performed (Zetasizer Nano ZS, Malvern). The zeta potential was estimated from electrophoretic mobility by using the Henry equation: *U*_E_ = 2εζ*F*(*ka*)/3η, where *U*_E_ is the electrophoretic mobility, ζ the zeta potential, ε the dielectric constant, *F*(*ka*) Henry’s function (set for 1.5 as in the Smoluchowski approximation), and η the viscosity. The pH value was adjusted with 0.1 mol/L of HCl or NaOH solutions. On the basis of the obtained results, the value of the isoelectric point (IEP) was determined by interpolating the ζ potential to zero for each of the tested samples.

Surface superoxide species were identified by using electron paramagnetic resonance (EPR) spectroscopy. Niobia samples treated with H_2_O_2_ and dried in air were outgassed and sealed in quartz tubes. Powder EPR spectra were measured with a Bruker Elexsys E580 spectrometer after quenching in liquid nitrogen. The modulation amplitude of 0.1 mT was used with 10 mW microwave power.

Surface peroxo species were detected by Raman spectroscopy. For this purpose, a Renishaw inVia dispersion spectrometer equipped with a CCD detector and coupled with a Leica DMLM confocal optical microscope was used. The laser excitation wavelength of 785 nm was used, and the laser power at the sample position was adjusted to 1.5 mW.

Free radicals (ROS) generated in the solution were detected in the spin trapping experiments. The resulting EPR spectra were recorded with a Magnettech MS400 spectrometer. In a typical experiment, 0.5 mg of Nb_2_O_5_ was added to a solution composed of 0.5 mL of DMPO solution (4 mg/1 mL) and 0.25 mL of 1.2% H_2_O_2_. In the case of H_2_O_2_-pretreated niobia, 0.5 mg of Nb_2_O_5_/H_2_O_2_ was added to 0.5 mL of DMPO solution and diluted to the same final volume. The aqueous sample was then transferred to a quartz capillary, and the EPR spectra were recorded at room temperature. The EPR parameters of the obtained spin adducts and surface species (powder EPR spectra) were determined by computer simulations of the experimental spectra by using EPRsim32 software.^29^

### 2.4 CIP Removal Studies

All reactions were performed in the dark at 25 °C with the use of an EasyMax 102 Advanced Thermostat system (Mettler Toledo). In a typical experiment, 50 mg of the catalyst was added to 100 mL of CIP solution (*C*_CIP_ = 15 mg/L) under vigorous stirring. After a given reaction time, CIP removal was estimated on the basis of UV–vis measurements. For this purpose, 4 mL aliquots were collected from the reactor, and the catalyst was separated from the solution by filtration through a 0.2 μm Whatman filter (PTFE). The absorption spectrum of the filtrate was recorded by using a spectrophotometer (Varian Cary 300) in the range 200–400 nm. The CIP concentration in the catalytic tests ranged from 5 to 30 mg/L. In this concentration range, a linear correlation was observed between absorbance (λ_max_ = 270 nm) and CIP concentration (see Figure S2).

For catalytic tests performed in the presence of hydrogen peroxide, the latter was added to the CIP solution before the addition of niobia. As shown in Figure S3, the presence of a small amount of H_2_O_2_ in the reaction medium (from 10 to 100 μL of concentrated H_2_O_2_ per 100 mL of CIP solution) had a negligible influence on the absorbance of CIP at 270 nm. Therefore, the removal of CIP in the presence of H_2_O_2_ could be correctly estimated from UV–vis measurements. During experiments under acidic or alkaline conditions, the pH of the reaction mixture was adjusted to a given value before the addition of niobia with nitric acid or sodium hydroxide.

For regeneration experiments under UV irradiation, the reaction vessel was irradiated from the top with a 200 W Hg–Xe lamp (Hamamatsu LC8 spot light) equipped with a UV filter (transmissive light of around 365 nm only) and a UV-light guide (model: A10014-50-0110). In a typical reuse experiment, the catalyst was separated from the postreaction mixture by centrifugation and then used in the next reaction cycle without any additional treatment.

The degradation products formed during the adsorption of CIP in the presence of H_2_O_2_ and after regeneration with UV light were analyzed by using the liquid chromatography mass spectrometry (LC-MS) technique. The degradation products were separated in an UltiMate TM 3000 UHPLC system (Thermo Scientific/Dionex) using a Kinetex C18 column (100 × 2.10 mm i.d., 2.6 μm, Phenomenex). The mobile phase consisted of water and methanol containing 0.1% formic acid (v/v), and the flow rate was set to 0.4 mL/min. The gradient started with a 10% methanol solution and increased to 20% in 8 min and to 45% in 7 min; this composition was maintained for 2 min, and then the initial composition was restored in 2 min, followed by an equilibration time of 5 min. The column was kept at 30 °C. The mass spectra were recorded by using a hybrid QTOF instrument (Impact HD, Bruker Datonics). It was operated in positive ion mode by using ESI under the following conditions: end plate voltage 500 V, capillary voltage 4.2 kV, nebulizer pressure 1.5 bar, dry gas (nitrogen) temperature 200 °C, dry gas flow rate 8 L/min. The spectrometer was previously calibrated with the standard tune mixture for the *m*/*z* range 100–500. Corroborative measurements of the total organic carbon (TOC) content were performed by using a total organic carbon analyzer (TOC-L, Shimadzu, Japan).

The antibacterial activity of CIP solutions after treatment with Nb_2_O_5_ in the presence of H_2_O_2_ or Nb_2_O_5_/H_2_O_2_ was examined by using the disc diffusion method with reference strains of *Bacillus subtilis* (ATCC 6633) and *Escherichia coli* (ATCC 10536). Mueller-Hinton Agar Petri plates (Merck Life Science, Poland) were first inoculated with 2 × 10^8^ cfu mL^–1^ cells to form a bacterial lawn. Then 20 mm Whatman grade 1 filter paper discs (GE Healthcare, Poland) were impregnated with 30 μL of the tested solution and placed separately on the agar plates. The experiment was performed in triplicate. Plates were incubated for 24 h at 37 °C, and the diameter of the inhibition halo was measured and used as an indicator of the residual antibacterial activity of the CIP degradation products.

The concentration of antibiotic adsorbed on the surface of the niobia samples was estimated on the basis of elemental analysis using a FLASH 2000 elemental analyzer (Thermo Scientific). Infrared spectra of niobia after CIP adsorption and regeneration with UV light were acquired by using a Bruker Vertex 70 spectrophotometer equipped with an attenuated total reflectance (ATR) accessory (Bruker). Before ATR-IR measurements, the samples were separated from the reaction mixtures by centrifugation and dried overnight at 60 °C.

## 3 Results and Discussion

### 3.1 ROS Formation after Interaction of H_2_O_2_ with Nb_2_O_5_

**Figure 1:**
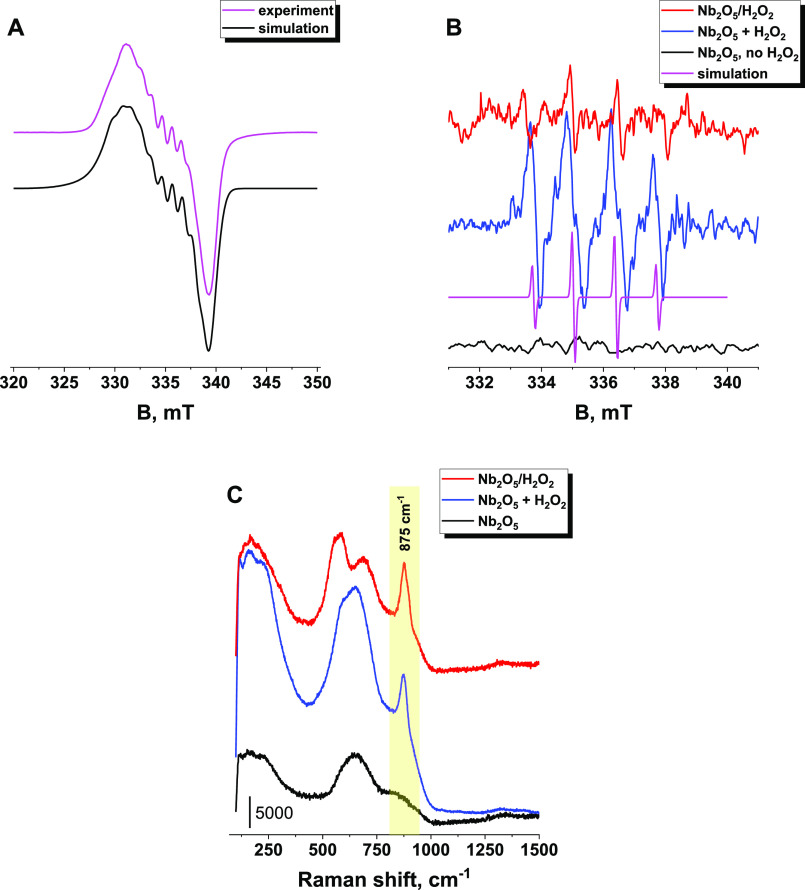
(A) Powder EPR spectrum of Nb_2_O_5_ pretreated with H_2_O_2_, (B) isotropic EPR spectra of solution samples after reaction of H_2_O_2_ with Nb_2_O_5_ in the presence of DMPO spin trap, and (C) Raman spectra of pristine Nb_2_O_5_ and Nb_2_O_5_ pretreated with H_2_O_2_.

To probe the products of the interaction of H_2_O_2_ with the surface of Nb_2_O_5_, EPR and Raman techniques were employed. Both the liquid and solid phases were analyzed. These measurements revealed a significant ability of amorphous Nb_2_O_5_ to generate surface superoxide radical anions (O_2_^•–^) and peroxo species (O_2_^2–^). The presence of O_2_^•–^ was confirmed by its characteristic EPR spectrum recorded for the solid phase dried after interaction with an aqueous solution of H_2_O_2_ (Figure 1A). Computer simulation of the resulting spectrum provided the following spin-Hamiltonian parameters: *g*_*xx*_ = 2.000, *g*_*yy*_ = 2.017, *g*_*zz*_ = 2.032, |*A*_*yy*_| = 1 mT, with *A*_*xx*_ and *A*_*zz*_ remaining unresolved. The powder EPR spectrum exhibits a rhombic signal with a resolved hyperfine structure (*A*_*yy*_) due to interaction with the ^93^Nb nucleus (*I* = 9/2, 100%). The EPR parameters obtained are indicative of the presence of the surface Nb(V)–O_2_^•–^ adducts, where the superoxide radical anions are directly bound to a center of Nb(V). Furthermore, EPR measurements of the liquid phase by using the DMPO spin trap revealed the presence of hydroxy radicals (^•^OH) (Figure 1B). Both Nb_2_O_5_ and Nb_2_O_5_/H_2_O_2_ were positively tested in this reaction; however, pristine Nb_2_O_5_ appeared to be more active in ^•^OH generation.

Furthermore, the formation of peroxo species was proven by the presence of the diagnostic Raman band at 875 cm^–1^ (Figure 1C). A more detailed account of the reactivity of Nb_2_O_5_ in the activation of hydrogen peroxide toward the formation of ROS has been described in our previous publications.^19,30^

### 3.2 Removal of CIP in the Presence of H_2_O_2_

**Figure 2:**
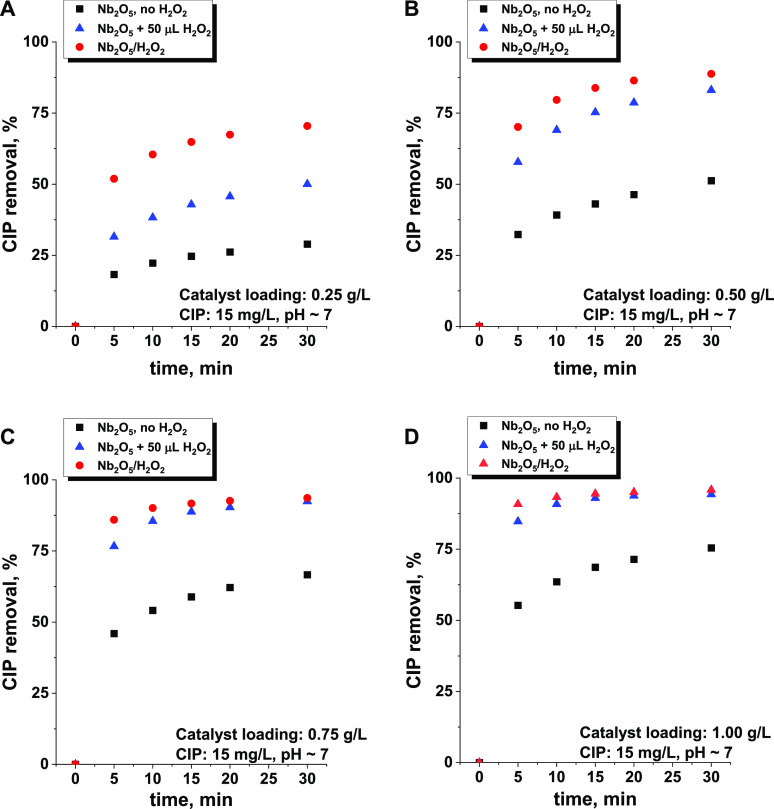
Effect of increasing catalyst loading [(A) 0.25, (B) 0.50, (C) 0.75, and (D) 1.00 g/L] on changes in CIP removal after interaction with Nb_2_O_5_ and Nb_2_O_5_ pretreated with H_2_O_2_. The reaction conditions are given in the graphs. All experiments were performed in the dark to avoid photocatalytic degradation of the pollutant.

To fully understand the role of ROS in the removal of CIP over the niobia catalyst, the following catalytic experiments were performed under various conditions: (i) in the absence of H_2_O_2_ in the reaction medium (no ROS is formed on Nb_2_O_5_), (ii) in the absence of H_2_O_2_ but with the use of the niobia catalyst treated previously with concentrated hydrogen peroxide and dried (superoxo and peroxo species were formed *ex situ* on the surface of Nb_2_O_5_ without the involvement of hydroxyl radicals), and (iii) in the presence of a small amount of H_2_O_2_ in the reaction medium (ROS is formed *in situ* on the surface of niobia during the reaction). The kinetic results of these experiments obtained for successively increasing loading of the niobia catalyst are shown in Figure 2.

The lowest efficiency of CIP removal was observed for the reaction performed in the absence of H_2_O_2_, for the process depended only on CIP adsorption without its chemical degradation. When a small amount of H_2_O_2_ was added to generate ROS *in situ*, the efficiency of CIP removal was significantly improved. The greatest response was observed for the lowest load of Nb_2_O_5_ (0.25 g/L). In this case, the amount of CIP removed increased nearly twice after 30 min. The highest efficiency was observed for the reaction with niobia pretreated with concentrated H_2_O_2_ (Nb_2_O_5_/H_2_O_2_). In this case, a further decrease in the CIP concentration by almost 50% (compared to that in the Nb_2_O_5_ + CIP + H_2_O_2_ system) was observed, but only for the lowest catalyst loading. For the highest loadings, after 30 min of reaction time, this progressive effect almost disappeared. The strongest influence of *in situ* or *ex situ* ROS formation on the elimination of CIP observed for 0.25 g/L catalyst loading was related to the lowest CIP removal efficiency (up to 75%) (Figure 2A). For higher loads (and higher conversion), the observed changes were still noticeable but much less pronounced (Figure 2B). When loadings of as high as 0.75 and 1.00 g/L were applied, the observed level of CIP removal was almost the same (for both native catalysts with H_2_O_2_ and Nb_2_O_5_ pretreated with H_2_O_2_), indicating that the equilibrium state was most likely achieved, resulting in depletion of ∼95% of the initial antibiotic concentration (Figure 2C,D).

Because at the highest catalyst loadings the changes in CIP removal after longer reaction times were negligible, the kinetics of the process was analyzed on the basis of the experiments with the use of the smallest concentration of the catalyst. As evidenced in Figure 2A, a high decrease in CIP concentration was achieved after 5 min of the process. Extending the reaction time from 5 to 30 min increased the efficiency of CIP removal by ∼20%. Consequently, there were no significant differences in the CIP removal rates with the use of parent Nb_2_O_5_ (no H_2_O_2_ and without ROS) and in the reaction in which ROS was present on the surface of the niobia (formed *in situ* or *ex situ*). Furthermore, a very fast decrease in CIP concentration at the beginning of the reaction (up to 5 min) and significantly less pronounced changes in the course of the reaction progress might suggest that the main process responsible for the elimination of CIP was simple adsorption. Given these primary observations, we hypothesized that the chemical degradation of CIP caused by ROS was negligible under the applied reaction conditions, while the adsorption of the antibiotic on the surface of the niobia played a major role in its removal.

**Figure 3:**
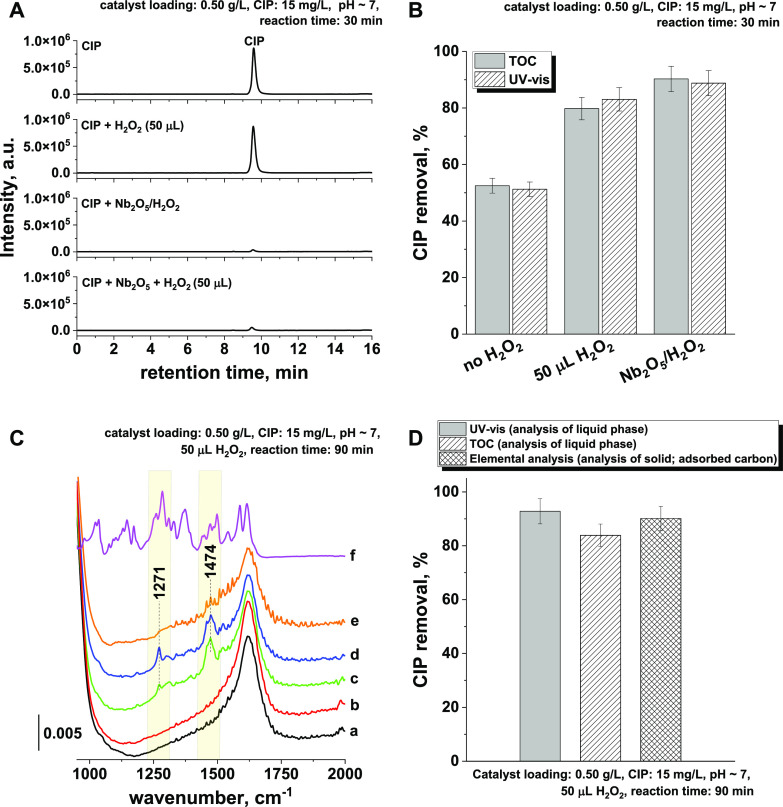
Analysis of CIP adsorption/degradation products. (A) LC-MS analysis of CIP and postreaction solutions, (B) comparison of CIP removal determined with UV–vis vs TOC, (C) ATR-IR spectra of adsorbed CIP on Nb_2_O_5_ (a, parent Nb_2_O_5_; b, Nb_2_O_5_ after interaction with H_2_O_2_; c, parent Nb_2_O_5_ after CIP adsorption (Nb_2_O_5_ + CIP); d, Nb_2_O_5_ after CIP adsorption in the presence of H_2_O_2_; e, Nb_2_O_5_ after CIP adsorption in the presence of H_2_O_2_ and after regeneration with UV irradiation; f, powder ciprofloxacin), and (D) comparison of the final CIP removal efficiency obtained with UV–vis, TOC, and elemental analysis of adsorbed antibiotic. All reactions were performed in the dark. Irradiation with UV light was applied only when indicated.

To verify this hypothesis, postreaction mixtures were analyzed with LC-MS, and the results are shown in Figure 3A. The chromatogram recorded for the initial CIP solution shows a single very intense peak at a retention time of 9.4 min and a second peak of very low intensity recorded at 8.5 min. On the basis of the corresponding mass spectra, the former peak was assigned to CIP molecules (see Figure S4), while the minute peak was attributed to some unidentified impurity present in negligible amounts (since no degradation products of CIP could be formed under such conditions; see Figure S5). Similar analysis of the LC-MS data obtained for the mixture of CIP solution and H_2_O_2_ revealed no changes in the chromatograms, indicating that the antibiotic could not be degraded by H_2_O_2_ in the absence of the catalyst. In the case of ROS formed *in situ* after the interaction of H_2_O_2_ with niobia or when niobia was pretreated with H_2_O_2_, the intensity of the CIP peak was negligible.

Consistent with the kinetic data, the highest decrease in CIP concentration was found for the reaction in which ROS was formed *ex situ* on the surface of the niobia (see Figures 3A and S6). Interestingly, no noticeable degradation products were observed either for the pretreated catalyst with Nb_2_O_5_/H_2_O_2_ or in the presence of H_2_O_2_ in the reaction mixture. It shows that under such conditions the rate of CIP degradation was much slower than that of antibiotic adsorption on the niobia surface. It is important to note that during mass spectrometry experiments the *m*/*z* range was scanned from 100 to 500. Thus, some degradation products with low *m*/*z* values could be overlooked. To resolve this issue, additional analyses of total organic carbon (TOC) in postreaction mixtures were performed.

As shown in Figure 3B, the data obtained from TOC analyzes are in very good agreement with the results of UV–vis measurements. This consistency supports the adsorptive mechanism of CIP removal. In fact, the IR spectra of the spent niobia catalysts separated from the reaction mixtures (Figure 3C) revealed the presence of typical ciprofloxacin bands (for example, 1474 and 1271 cm^–1^). CIP can be efficiently adsorbed in its parent form on the surface of the niobia, even in the presence of H_2_O_2_ and ROS. The amount of adsorbed CIP was assessed on the basis of elemental analysis (Figure 3D) and was found to be comparable to that calculated from the data on CIP removal from the liquid phase (UV–vis and TOC measurements). Thus, given all the analytical results, one can conclude that the enhanced removal of CIP in the presence of ROS on the surface of the niobia resulted mainly from the improved ability of this metal oxide to adsorb the antibiotic molecules.

**Figure 4:**
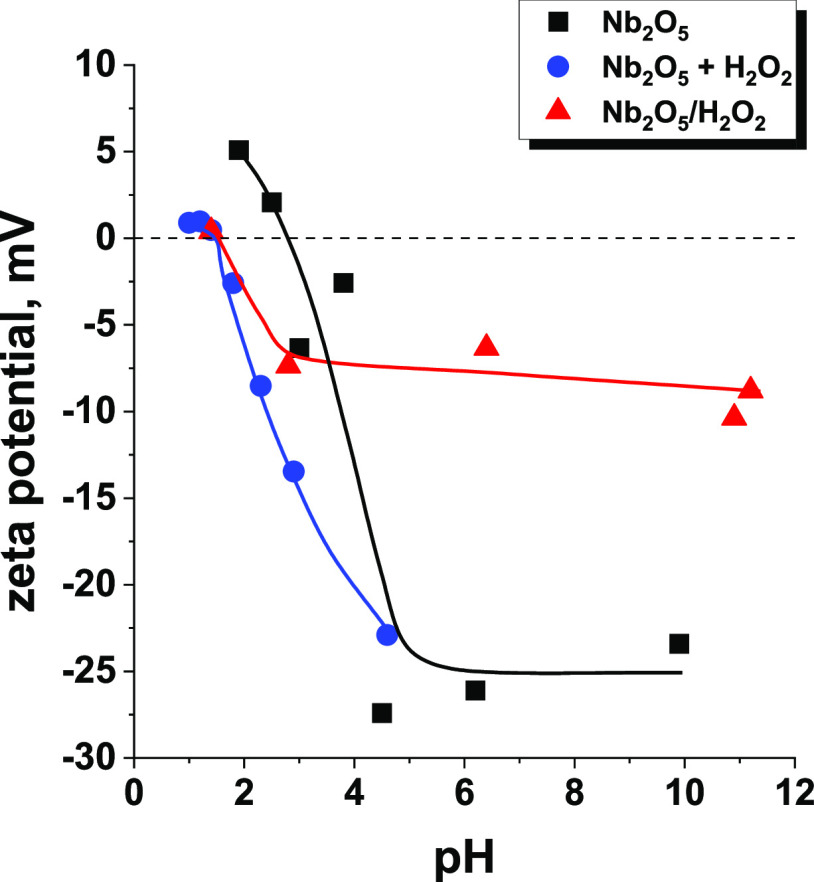
Influence of H_2_O_2_ on ζ potential measurements of amorphous Nb_2_O_5_ and Nb_2_O_5_ pretreated with hydrogen peroxide in the dark.

Because of the Brønsted acidity of the Nb_2_O_5_ surface and the zwitterionic nature of the CIP molecules, we further investigated the influence of pH on the adsorption and degradation process. At first, the total surface charge as a function of pH was established based on the results of the ζ potential measurements shown in Figure 4. The results obtained indicate a slight change in the isoelectric point of the parent Nb_2_O_5_ (IEP = 2.9) toward a lower value (1.5) after the accumulation of negatively charged ROS on the surface. For the sample Nb_2_O_5_/H_2_O_2_, the changes in ζ potential with pH are less abrupt than for Nb_2_O_5_ (also for niobia in the presence of H_2_O_2_ in solution), and the surface exhibits a buffering effect due to the presence of abundant surface ROS. Taking into account the above results, two different pH values were chosen, at which electrostatic repulsion between the surface of the niobia and the CIP molecules should result in very low adsorption: (i) pH ∼ 2.5 at which CIP exists in the form of protonated molecules^31^ and the surface of the parent niobia is positively charged; (ii) pH ∼ 10.5 at which the CIP molecules and the surface of the niobia are both negatively charged.^31^

**Figure 5:**
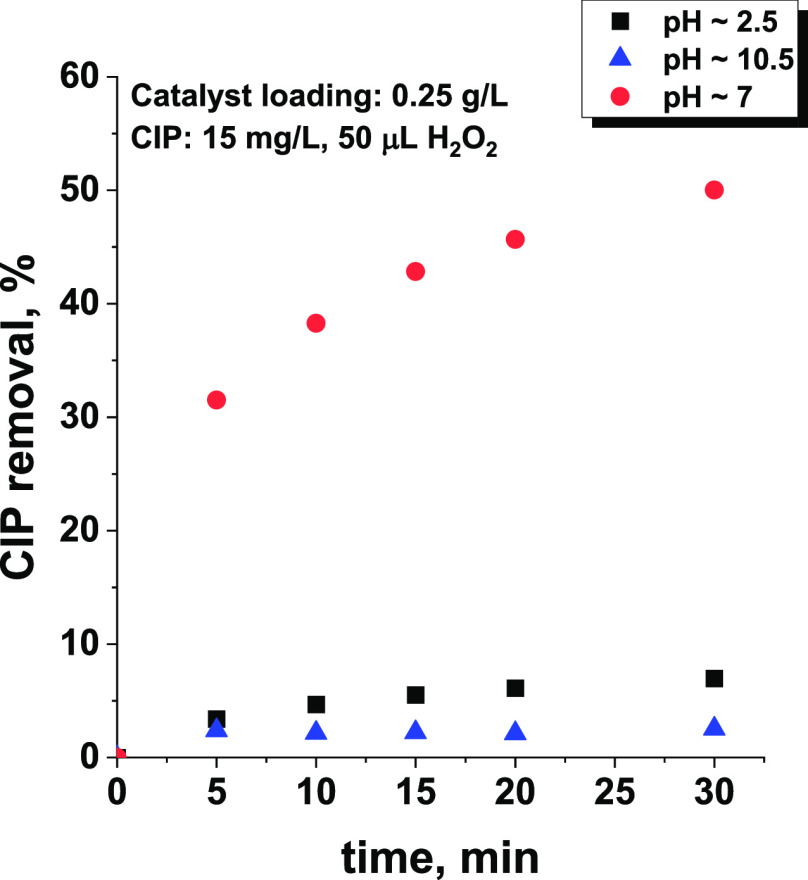
Influence of pH on the efficiency of CIP removal with Nb_2_O_5_. All reactions were performed in the dark to avoid photocatalytic degradation of the antibiotic.

**Figure 6:**
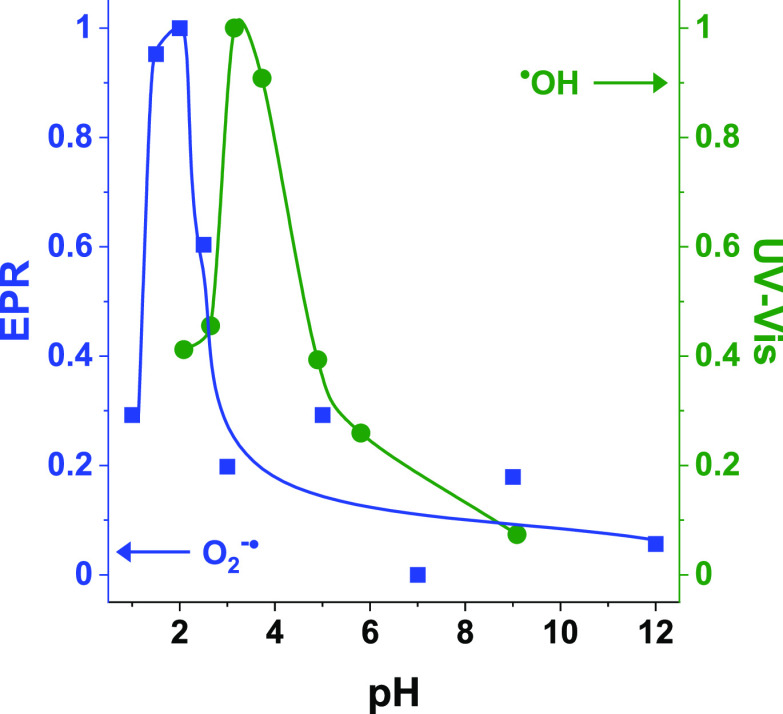
Influence of pH on relative concentration of ^•^OH and O_2_^•–^ radicals formed during the interaction of H_2_O_2_ with the parent amorphous Nb_2_O_5_.

Negligible antibiotic adsorption was expected to allow a precise determination of the contribution of the degradation process, especially under acidic conditions in which niobia shows a very low ability to adsorb antibiotics and a relatively high ability to activate H_2_O_2_ toward ROS formation.^19^ As shown in Figure 5, the removal of CIP under both highly alkaline and acidic conditions was negligible, indicating that the efficiency of antibiotic degradation by ROS formed on the surface of the niobia was very low in both cases. The slight differences observed at pH ∼ 2.5 and pH ∼ 10.5 resulted from the shift in the IEP value induced by H_2_O_2_ (see above). At pH close to 2.5 the surface becomes neutral or slightly negatively charged, which lowers the electrostatic barrier of adsorption. Therefore, the highest activity of Nb_2_O_5_ under neutral conditions (pH ∼ 7) in which the formation of hydroxyl radicals was found to be significantly less efficient than under acidic conditions (see Figure 6) must originate mainly from the adsorption process. Note that under neutral conditions the antibiotic existed in the zwitterion form,^31^ while the surface of the niobia was negatively charged (pH above the isoelectric point of Nb_2_O_5_, Figure 4). Under such conditions, CIP molecules could easily be adsorbed on the surface of the niobia by electrostatic interaction.^31,32^

**Figure 7:**
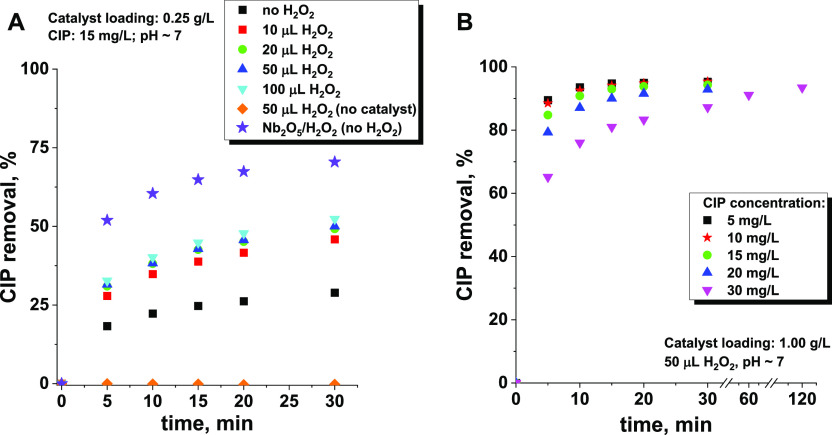
Changes in the efficiency of CIP removal with (A) H_2_O_2_ concentration and (B) CIP concentration in the reaction medium. All reactions were performed in the dark to avoid photocatalytic degradation of the antibiotic.

To gain a deeper understanding of the role of H_2_O_2_ and ROS in the removal of CIP in the presence of niobia, additional experiments were performed by using various concentrations of hydrogen peroxide. As shown in Figure 7A, the positive impact of hydrogen peroxide was noticeable even at a very low H_2_O_2_ dose of 10 μL per 100 mL of CIP solution. A further increase in H_2_O_2_ doses from 10 to 100 μL enhanced slightly improved antibiotic adsorption, but this effect was still much less pronounced than that observed for the H_2_O_2_ pretreated niobia catalyst in which ROS were formed *ex situ*. This observation is well correlated to the amount of surface ROS detected for Nb_2_O_5_ contacted with the H_2_O_2_ solution and the Nb_2_O_5_/H_2_O_2_ sample. For the latter, the estimated amount of ROS was 5 times higher (Figure 1C).

In addition, we evaluated the impact of CIP concentration on the reaction in which ROS were formed *in situ* on the niobia surface. The results are shown in Figure 7B. Very high efficiency was observed within the initial 5 min of the adsorption process, almost in the entire concentration range studied (5–30 mg/L). Only in the case of the highest concentration was CIP depletion slightly reduced in a short reaction time, but it was significantly increased when the reaction time was extended. After 2 h of the process, more than 90% of the CIP was successfully removed. Therefore, the results obtained above from the activity tests are representative of the investigated CIP concentration range, not only for the selected value.

**Figure 8:**
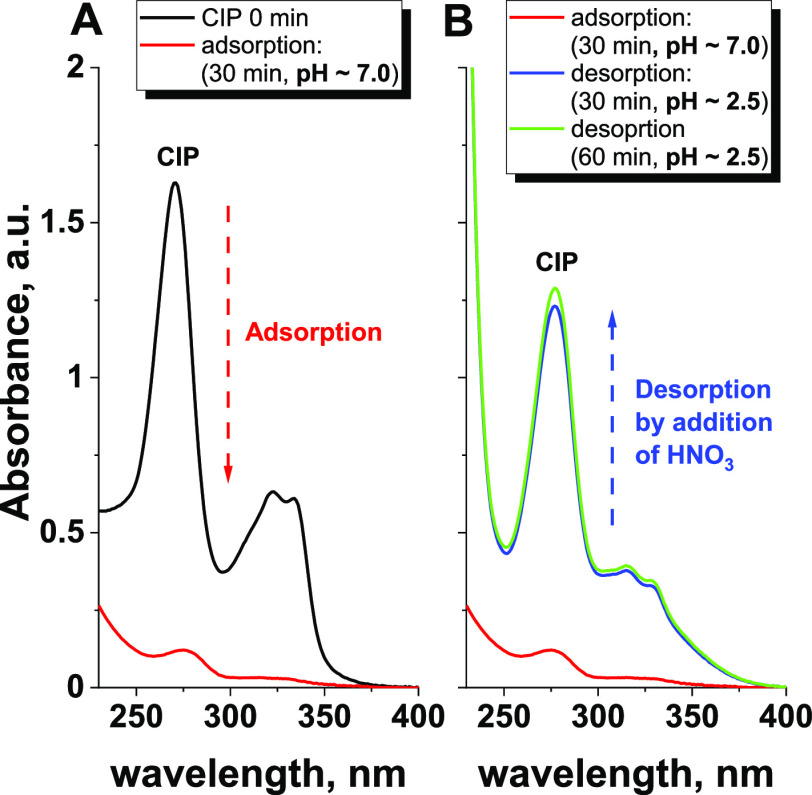
UV–vis spectra showing (A) CIP adsorption under neutral conditions and (B) CIP desorption after acidification of the reaction medium. Reaction conditions: catalyst loading = 1.00 g/L, CIP = 15 mg/L, 50 μL of H_2_O_2_. All reactions were performed in the dark to avoid photocatalytic degradation of the antibiotic.

The results of CIP removal at different pH levels (Figure 5) clearly show that the antibiotic cannot be efficiently adsorbed on the surface of the niobia when the pH of the reaction mixture is strongly acidic or alkaline. Given this observation, we expect that the change in pH of the reaction mixture after the adsorption step can be used for the desorption of CIP molecules from the niobia surface and regeneration of the spent catalyst. Indeed, by using a niobia catalyst preadsorbed with CIP under neutral conditions in the presence of H_2_O_2_ and then decreasing the pH of the reaction mixture to 2.5, we observed reversible adsorption–desorption cycles (Figure 8A,B).

During the adsorption step (Figure 8A), more than 90% of the initial CIP molecules were removed from the solution. Already after the addition of 2 droplets of concentrated nitric acid to the reaction mixture (pH changed from 7.0 to 2.5), we observed a significant desorption of CIP from the catalyst (Figure 8B). These results not only demonstrate that CIP adsorbed on the niobia surface can be simply desorbed by changing the pH but also unambiguously indicate that CIP molecules were not degraded under such reaction conditions. Regeneration of the niobia catalyst by adjusting the pH requires further optimization, but these initial results clearly show that ROS formed on the surface of the niobia are responsible mainly for its enhanced ability to adsorb CIP molecules.

### 3.3 Regeneration of Nb_2_O_5_ Catalyst with UV Light

**Figure 9:**
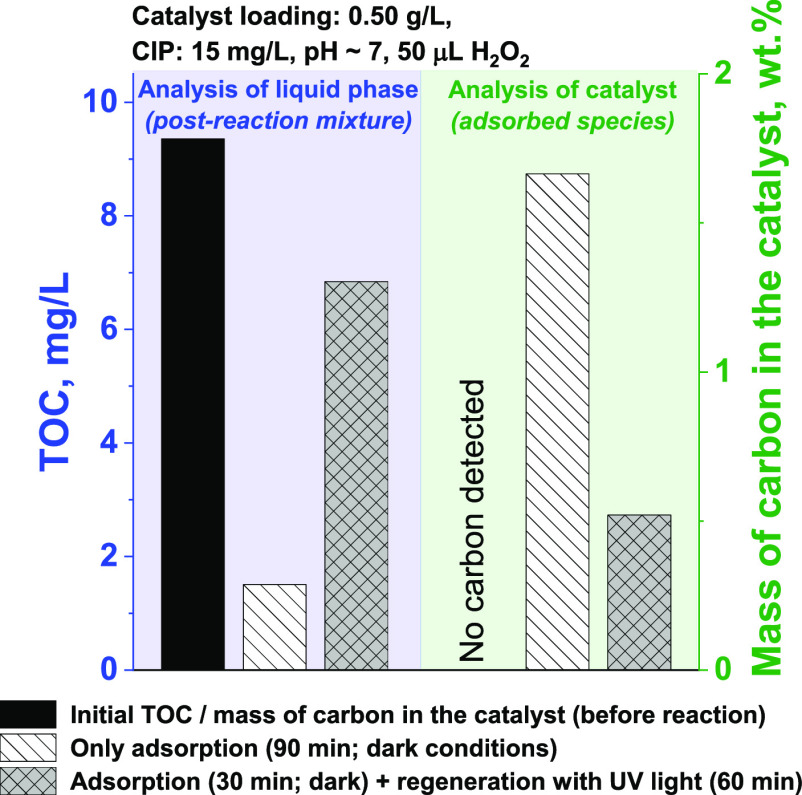
Analysis of organic components dissolved in the liquid phase and adsorbed on the surface of the niobia as a result of the interaction of CIP and H_2_O_2_ with Nb_2_O_5_ in the dark (adsorption step) and after UV irradiation (regeneration step).

**Figure 10:**
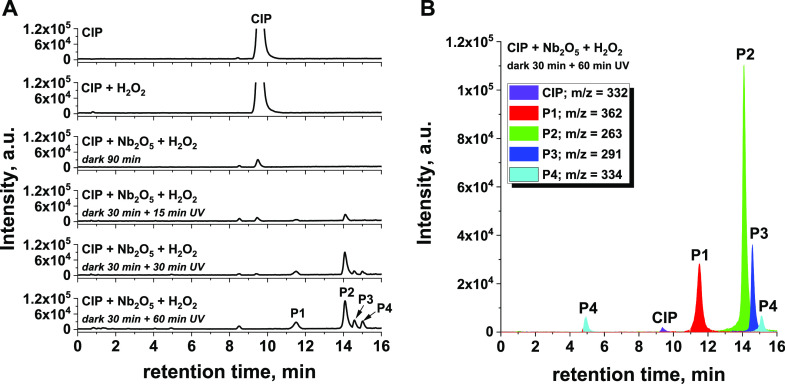
(A) Total ion chromatograms of the postreaction media of the CIP, H_2_O_2_, and Nb_2_O_5_ system interacting in the dark or under UV irradiation. (B) Extracted ion mass chromatogram of the postreaction mixture after 30 min of the reaction in the dark followed by 60 min of UV irradiation obtained by LC-ESI(+)-MS. Reaction conditions: catalyst loading = 0.50 g/L, CIP = 15 mg/L, pH ∼ 7, 50 μL of H_2_O_2_. Irradiation with UV light was applied only when indicated.

A recent study by Taher et al.^17^ has shown that the photocatalytic properties of Nb_2_O_5_ can also be used for the regeneration of spent niobia catalysts after adsorption of methylene blue. Inspired by this idea, we not only investigated the influence of UV irradiation on the spent niobia samples but also performed a detailed analysis of the degradation products that could be formed in response to exposure of Nb_2_O_5_ to both H_2_O_2_ and light. To do this, the reaction mixture was stirred in the dark for 30 min (adsorption step) and then irradiated for 60 min with UV light (λ = 365 nm; regeneration step). A control reaction performed in the dark for 90 min was performed in parallel. Particular attention was paid to the analysis of both organic species adsorbed on the surface (elemental analysis and ATR-IR) and organic molecules in the liquid phase (LC-MS and TOC). The results of these experiments are summarized in Figures 9 and 10.

After 90 min of reaction in the dark, the concentration of organic species adsorbed on the surface of the niobia in the presence of H_2_O_2_ increased significantly (Figure 9). At the same time, no noticeable degradation products were identified by LC-MS (compare Figure 10A), which confirmed the adsorption of CIP only under such conditions. Interestingly, when the reaction mixture was irradiated with UV light (regeneration step), the amount of TOC in the liquid phase increased significantly at the expense of the organic species adsorbed on the surface of the niobia (Figure 9). It is important to emphasize that the decrease in the concentration of organic species adsorbed on the surface of the niobia (estimated by elemental analysis) and the increase in the total organic carbon (TOC) in the liquid phase were commensurate.

The observed changes resulted from the degradation of adsorbed CIP molecules upon UV irradiation, forming less complex organic compounds that were then desorbed from the niobia surface. Indeed, LC-MS analyses of the postreaction mixtures clearly showed that after the UV regeneration step several new peaks appeared in the chromatograms (Figure 10A). A more detailed analysis of the mass spectra based on the observed values of *m*/*z* detected by LC-MS allowed identification of the degradation products (Figure 10B and Table 1; mass spectra of the identified compounds are shown in Figure S7). In particular, exposure to UV light was found to result in degradation of the piperazine moiety of the CIP molecule to produce P2 as the main product. According to the literature,^33^ this degradation pathway indicates a predominance of the surface reaction mechanism in which CIP molecules adsorbed on the photocatalysts are oxidized by photogenerated holes (h^+^). This degradation mechanism is in agreement with that previously established for niobia-based photocatalysts, in which photogenerated holes have been identified as the main oxidative species responsible for the degradation of selected organic pollutants.^34^ Taking into account the amorphous nature of the investigated Nb_2_O_5_, which can suppress effective photocatalytic activity, it cannot be excluded that degradation of CIP molecules during the UV regeneration step proceeded, to some extent, by simple photolysis of the antibiotic, as pointed out elsewhere.^35^

**Table 1:**
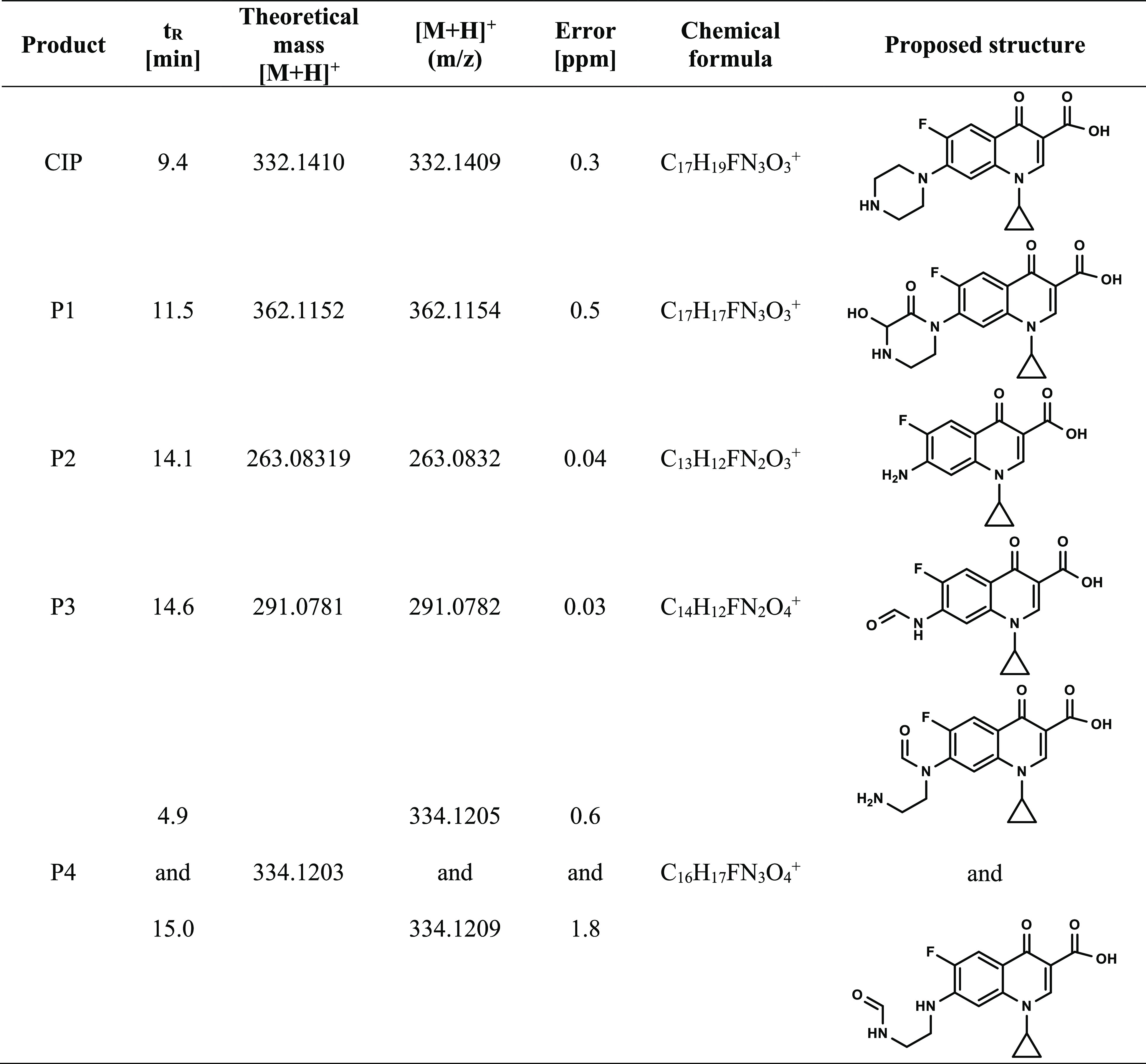
CIP and Its Degradation Products Identified by LC-MS Analysis of the Postreaction Mixtures after 30 min of Reaction in the Dark (Adsorption Step) Followed by 60 min of Irradiation with UV Light (Regeneration Step)

Therefore, analysis of the LC-MS data, in combination with the results obtained from the elemental analysis and TOC measurements, allowed us to conclude that the piperazine moiety was probably responsible for the efficient adsorption of CIP on the Nb_2_O_5_ surface. Upon degradation of this functional group, the formed organic pollutants P1–P4 (Table 1) were desorbed from the metal oxide, leading to regeneration of the spent catalysts.

Such a process was also indicated by ATR-IR studies. As shown in Figure 3C, the typical vibrational bands of CIP molecules adsorbed on the niobia could be easily identified in the IR spectra of the spent solid samples, which were not irradiated with UV light (spectrum d). However, after irradiation with UV light, the typical IR bands disappeared (spectrum e). It should be noted that some IR bands typical of other organic compounds could still be detected, indicating their presence on the niobia surface after the regeneration step. These results are in agreement with the data obtained from the elemental analysis showing that some organic species were still present on the surface of the niobia after 60 min of UV irradiation (Figure 9).

**Figure 11:**
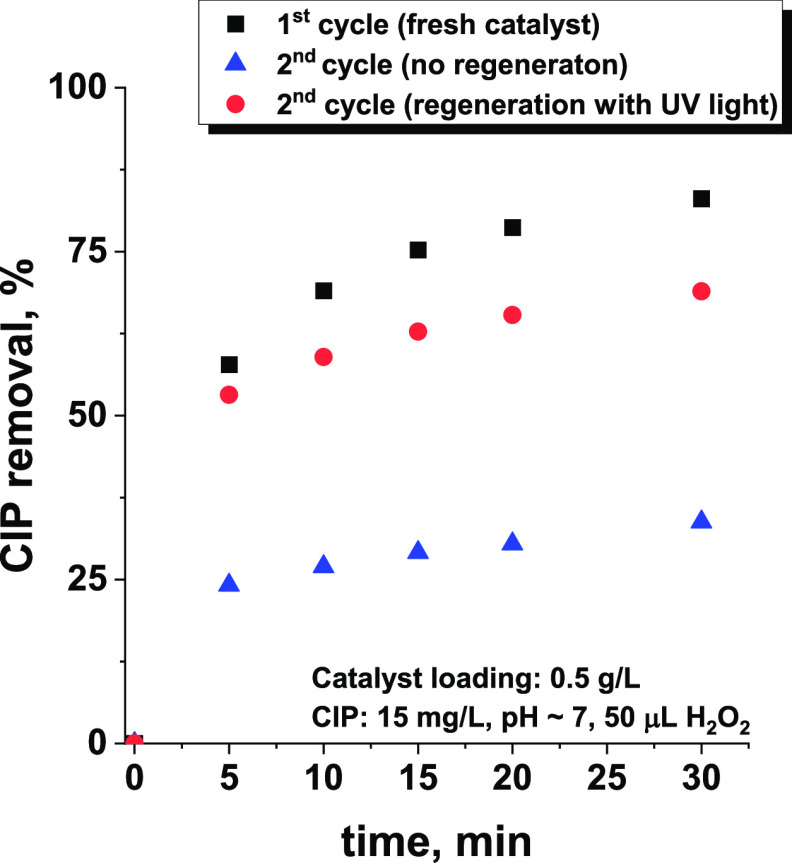
Comparison of efficiency of CIP removal for fresh Nb_2_O_5_ sample (first cycle), used sample (second cycle), and sample after first cycle regenerated by irradiation with UV light for 60 min. The reaction was performed in the dark.

To investigate the possible recyclability of the niobia catalyst, we used regenerated oxide in a second reaction cycle. As shown in Figure 11, the regenerated Nb_2_O_5_ removed a significantly higher amount of CIP than the sample not exposed to UV light (reference material that was stirred in the dark for 90 min). The efficiency of CIP removal by the regenerated sample was slightly lower than that observed in the first reaction cycle by using the parent metal oxide, but the positive impact of regeneration could still be easily observed. The apparent decrease in efficiency compared to that of the fresh sample is a result of the persistent presence of some CIP degradation products on the niobia surface. Such products occupying adsorption sites decreased the overall number of active sites available for CIP molecules. The presence of partially degraded products on the niobia surface was confirmed by ATR-IR measurements (see Figure 3C, spectra d and e) and elemental analysis (Figure 9). Therefore, niobia can be successfully regenerated upon exposure to light and hydrogen peroxide, but further studies are necessary to optimize the regeneration conditions to improve the mineralization efficiency of the antibiotic.

### 3.4 Biological Activity of CIP–Nb_2_O_5_ Assay

To further illustrate the great potential of Nb_2_O_5_ as a regenerable adsorbent for CIP removal, microbiological tests were performed to detect the cytotoxicity of antibiotic degradation products, formed during the UV regeneration step. Antibacterial activity toward *E. coli* and *B. subtilis* as model microorganisms was evaluated by using a disc diffusion method. As evidenced in Table 2, the initial CIP solution strongly inhibited the growth of both model bacteria. As expected, the inhibition effect was greatly reduced in the presence of the postreaction mixture obtained after the adsorption step (reaction in the dark), where most of the CIP was adsorbed on the surface of the niobia, but some amount of antibiotic was still detected in the reaction medium (as concluded from the LC-MS studies; see Figure 10A). No inhibition effect of bacteria growth was observed for postreaction mixtures after 60 min of the UV regeneration step, in which CIP was not identified by LC-MS, while the main degradation product was P2 (see Figure 10B and Table 1). Therefore, the antibacterial activity of CIP can be simply eliminated by degradation of the piperazine moiety upon exposure of the reaction mixture to UV light. It shows not only that niobia can be used for efficient elimination of antibiotics by adsorption but also that the spent niobia adsorbent can be easily regenerated in an environmentally benign way, leading to the formation of degradation products that do not exhibit any antibacterial activity toward *E. coli* and *B. subtilis*.

**Table 2:** Antimicrobial Activity of CIP and Its Degradation Products Tested against *E. coli* and *B. subtilis*^a^

	diameter of inhibition zone [mm]			
sample	*E. coli*	SD^d^	*B. subtilis*	SD^d^
control (saline)	20.00	0.00	20.00	0.00
CIP^b^	32.90	0.49	28.98	0.32
CIP + H_2_O_2_^b^	33.18	1.17	29.93	1.38
Nb_2_O_5_ + CIP + H_2_O_2_^b^	21.48	0.04	20.00	0.00
Nb_2_O_5_ + CIP + H_2_O_2_ + UV^c^	20.00	0.00	20.00	0.00

a Selected images presenting results of the diffusion tests are shown in Figure S8. Reaction conditions: 100 mL of CIP solution (15 mg/L), pH ∼ 7, 50 μL of H_2_O_2_ (30%), 50 mg of Nb_2_O_5_, 600 rpm, 25 °C.

b Stirred in the dark for 90 min.

c Stirred in the dark for 30 min followed by irradiation with UV light (λ = 365 nm) for 60 min (adsorption followed by regeneration).

d SD stands for standard deviation.

### 3.5 Role of ROS in Enhanced Adsorption of CIP on Nb_2_O_5_

Elimination of water-soluble organic pollutants requires the concerted action of adsorption and degradation by oxidation, which defines the so-called adsorption-triggered process.^36,37^ There have been several reports indicating that ROS formed after the interaction of niobia with H_2_O_2_ are highly active in the oxidative degradation of organic dyes^38^ and the selective oxidation of alcohols^28,39^ or olefins.^40^ However, the implicit contribution of ROS in the adsorption of organic pollutants on the niobia surface has not been considered yet. In this study, we have documented that ROS stabilized on the surface of niobia has a significant impact on the ζ potential of Nb_2_O_5_. For the parent niobium pentoxide, a strong negative charge immediately accumulated on the surface when the pH increased slightly above the isoelectric point of Nb_2_O_5_. The higher the pH value, the stronger the negative charge accumulated on the surface of the niobia (Figure 4). On the contrary, the ζ potential of niobium pentoxide treated with concentrated H_2_O_2_ was not as sensitive to an increase in the pH value. The results show that the surface of the Nb_2_O_5_/H_2_O_2_ catalyst was negatively charged at a pH value already lower than the IEP of the parent niobia, but the accumulated charge was not as strong as that established for Nb_2_O_5_. The negative charge did not change significantly when the pH value increased from 3 to 12, indicating very good stability of the suspension obtained in a wide pH range. It shows that the formation of ROS on the niobia surface caused some important changes in the properties of this metal oxide, preventing the accumulation of a strong negative charge on its surface. Previous studies by Ziolek et al.^19^ revealed that treatment of Nb_2_O_5_ with a H_2_O_2_ solution led to a significant decrease in the concentration of Brønsted acid sites (that is, acidic hydroxyl groups). On the basis of these results and given direct proofs of ROS accumulation and stabilization on the surface of Nb_2_O_5_ (Figure 1), we attribute the different behavior of the sample treated with hydrogen peroxide to the exchange of surface hydroxyl groups into superoxide radical anions or peroxo species. The more efficient the exchange, the lower the concentration of the surface hydroxyls, which could be deprotonated upon increasing the pH value. Therefore, we have established that the surface of the niobia covered with ROS, which is formed at the expense of surface hydroxyls, acted as a buffer, preventing the accumulation of a strong negative surface charge at pH values higher than the IEP of the parent Nb_2_O_5_.

**Figure 12:**
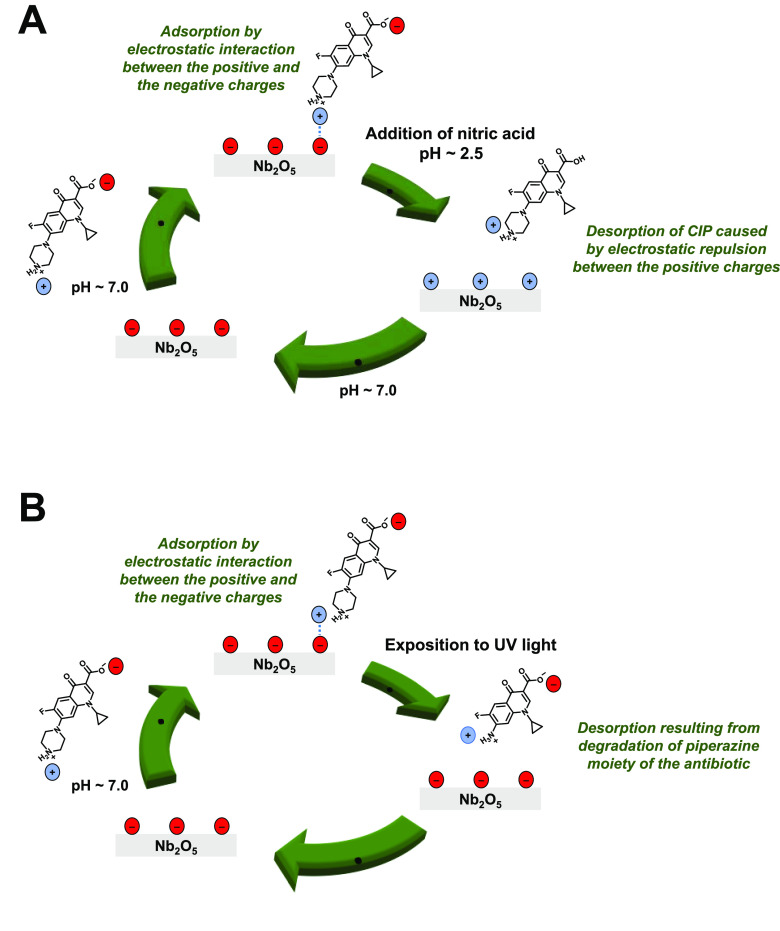
Possible CIP adsorption states and two different ways of regeneration of the Nb_2_O_5_ surface subjected to CIP and H_2_O_2_: (A) regeneration under acidic conditions; (B) regeneration by UV irradiation.

The results obtained in this study are in favor of the electrostatic interaction between the negatively charged surface groups of niobia and the positively charged nitrogen atom of the piperazine moiety of CIP, as shown schematically in Figure 12A. The highest CIP adsorption efficiency was observed under neutral conditions in which the antibiotic was present in zwitterionic form.^31^ Taking into account that such a form contains two opposite charges in two different functional groups, it is highly probable that the accumulation of a strong negative charge on the surface of the niobia may be responsible for the lower ability of the parent Nb_2_O_5_ to absorb CIP molecules. On the basis of experimental data, we claim that the strong negative charge on the surface of the parent Nb_2_O_5_ caused electrostatic repulsion between the surface and the negatively charged carboxyl group of CIP, and this resulted in a lower efficiency of CIP removal by the adsorption process than that observed for the niobia catalyst treated with hydrogen peroxide. The ζ potential measurements clearly showed that the latter samples exhibited a lower surface negative charge, providing more favorable conditions for the efficient adsorption of the antibiotic in the zwitterionic form.

As far as CIP adsorption is concerned, it is important to emphasize that adsorbed CIP molecules could be simply desorbed from the surface of the niobia by changing the pH of the reaction mixture (Figure 8B) or by degradation of the piperazine moiety of the antibiotic after irradiation with UV light (Figures 9 and 10). The adsorption of CIP on the niobia surface in the dark and regeneration of the spent niobia catalyst after exposure to UV light are schematically summarized in Figure 12.

Our studies clearly indicated that the adsorption of CIP on the niobia surface was caused by an electronic interaction between the protonated piperazine moiety of CIP and the negatively charged surface of niobia. This conclusion was supported by the desorption of the degraded CIP molecules when the piperazine moiety was oxidized upon exposure of the reaction mixture to UV light (Figure 10 and Table 1) and ineffective adsorption of CIP when the surface of Nb_2_O_5_ was positively charged at pH ∼ 2.5 (Figures 5 and 8). The main source of negative charge on the surface of the parent Nb_2_O_5_ is dissociated hydroxyl groups (dissociation of surface hydroxyls S–OH to S–O^–^ is observed at a pH above the IEP of Nb_2_O_5_). The same hydroxyl groups can be quantified by pyridine adsorption. According to our previous studies,^19^ the concentration of Brønsted acid sites in commercial Nb_2_O_5_ CBMM is equal to 100 μmol g^–1^. This value has been considered by us as a number of possible active sites that can take part in the adsorption of CIP molecules. On the basis of this, we estimate the number of active sites that were occupied by CIP molecules under various reaction conditions (Table 3). After 5 min of the reaction in the absence of hydrogen peroxide, ∼33% of the available adsorption sites were occupied by CIP molecules. When a small amount of H_2_O_2_ was added to the reaction mixture, the number of occupied adsorption sites increased from 33% to 57%. The highest number of possible adsorption sites was occupied when the reaction was performed in the presence of the Nb_2_O_5_/H_2_O_2_ catalyst. In this case, ∼94% of the initial sites were occupied by antibiotic molecules, but after 30 min of the reaction the calculated amount of the occupied sites exceeded 100%. This estimate clearly shows that CIP molecules were adsorbed not only on the deprotonated surface hydroxyl groups (S–O^–^) but also on negatively charged ROS stabilized on Nb_2_O_5_/H_2_O_2_ (e.g., superoxide and peroxide anions detected with EPR and Raman, respectively). Because no oxidative degradation of CIP was observed (reaction in the dark), the data collected in Table 3 confirm that treatment of Nb_2_O_5_ with H_2_O_2_ results in the formation of additional adsorption sites for CIP molecules.

**Table 3:** Calculation of the Amount of Adsorption Sites Derived from Brønsted Acid Centers and Their Occupancy Changes during Removal of CIP

		no. of CIP molecules adsorbed after given reaction time^c^ [mol]		amount of available adsorption sites occupied by CIP molecules after given reaction time [%]	
sample^a^	no. of available adsorption sites^b^ [mol]	5 min	30 min	5 min	30 min
Nb_2_O_5_	2.50 × 10^–6^	8.28 × 10^–7^	1.31 × 10^–6^	33	52
Nb_2_O_5_ + H_2_O_2_ (50 μL)	2.50 × 10^–6^	1.43 × 10^–6^	2.26 × 10^–6^	57	91
Nb_2_O_5_/H_2_O_2_	2.50 × 10^–6^	2.35 × 10^–6^	3.19 × 10^–6^	94	128

a Reaction conditions: 100 mL of CIP solution (15 mg/L), pH ∼ 7, catalyst loading: 0.25 g/L, reaction in the dark.

b Number of Brønsted acid sites in the parent Nb_2_O_5_ calculated form IR spectra after pyridine adsorption.^19^

c Calculated on the basis of CIP removal efficiency in the dark conditions.

## 4 Conclusions

In this paper, the contribution of adsorption and degradation processes to the removal of ciprofloxacin by amorphous niobium pentoxide in the presence of hydrogen peroxide was analyzed. Particular attention was paid to the evaluation of the role of reactive oxygen species (formed by the interaction of H_2_O_2_ with Nb_2_O_5_) in the elimination of the antibiotic in the dark. In addition to the well-established oxidative properties of surface ROS, widely used for the degradation of organic pollutants, it was found that the improved efficiency of Nb_2_O_5_ resulted from its improved ability to adsorb CIP molecules due to the formation of superoxo and peroxo species that modified the surface properties. At low catalyst loadings and with a short reaction time, adsorption was the main process responsible for the enhanced CIP elimination under dark conditions, while the contribution of the degradation by ROS was negligible. This unexpected role of ROS stabilized on the Nb_2_O_5_ surface needs to be emphasized because in many studies devoted to the removal of environmental pollutants with the use of niobia-based catalysts, the analysis of the adsorption contribution, which may be dominant in short reaction times, has been neglected. Instead, only oxidative degradation of organic compounds has been taken into account, which can lead to misinterpretation of the kinetic results and wrong conclusions about the reactivity of ROS in degradation processes. Furthermore, Nb_2_O_5_ appeared as a versatile catalytic material active in the adsorptive removal of CIP; the oxide can be easily regenerated by environmentally benign UV light treatment in the presence of H_2_O_2_ which results in degradation of CIP molecules and desorption of its degradation products from the niobia surface.

## Supporting Information Available

BET surface area and nitrogen physisorption data for commercial Nb_2_O_5_ CBMM, UV–vis spectra of CIP solutions, ESI-MS spectra and LC-MS analysis of CIP solutions and CIP degradation products, images of samples subjected antimicrobial tests (PDF)
