# Supplementary material for: Unraveling the Origin of Enhanced Activity of the Nb2O5/H2O2 System in the Elimination of Ciprofloxacin: Insights into the Role of Reactive Oxygen Species in Interface Processes
Source: ACS Appl Mater Interfaces. 2022 Jul 11;14(28):31824–37. doi: 10.1021/acsami.2c04743 (PMC9305982; doi:10.1021/acsami.2c04743)
Supplement: Supplementary file 1 — am2c04743_si_001.pdf [file am2c04743_si_001.pdf]

*Supporting Information*

# Unraveling the origin of enhanced activity of $Nb_2O_5/H_2O_2$ system in the elimination of ciprofloxacin: insights into the role of reactive oxygen species in interface processes

Lukasz Wolski[1,*], Kamila Sobańska[2], Malwina Muńko[3], Adrian Czerniak[3], Piotr Pietrzyk[2,*]

[1] *Faculty of Chemistry, Adam Mickiewicz University, ul. Uniwersytetu Poznańskiego 8, 61-614 Poznań, Poland*

[2] *Faculty of Chemistry, Jagiellonian University, ul. Gronostajowa 2, 30-387 Kraków, Poland*

[3] *Center for Advanced Technology, Adam Mickiewicz University, ul. Uniwersytetu Poznańskiego 10, 61-614 Poznań, Poland*

* Corresponding authors: wolski.lukasz@amu.edu.pl (L.W.), piotr.pietrzyk@uj.edu.pl (P.P.)

**Table of content:**

**Figure S1.** **(A)** Nitrogen adsorption-desorption isotherm and **(B)** pore size distribution estimated for commercial $Nb_2O_5$ CBMM catalyst. ....................S2

**Figure S2.** **(A)** UV-vis spectra of ciprofloxacin solution of different concentration. **(B)** Relationship between concentration of the antibiotic and its absorbance at $\lambda_{max}$ = 270 nm. ....................S3

**Figure S3.** Influence of $H_2O_2$ addition on estimation of antibiotic absorbance at $\lambda_{max}$ = 270 nm. .........S4

**Figure S4.** The ESI-MS spectrum of the chromatographic peak at 9.4 min, typical of ciprofloxacin. .S5

**Figure S5.** The ESI-MS spectrum of the chromatographic peak at 8.5 min....................S6

**Figure S6.** LC-MS analyses of CIP and post-reaction solutions shown at higher magnification. Full scale chromatograms are shown in Fig. 3A (see main text of the article)....................S7

**Figure S7.** The ESI-MS spectra of identified degradation products....................S8

**Figure S8**. Selected images presenting results of antimicrobial tests against **(A)** *E. Coli* and **(B)** *B. Subtilis*. ....................S9

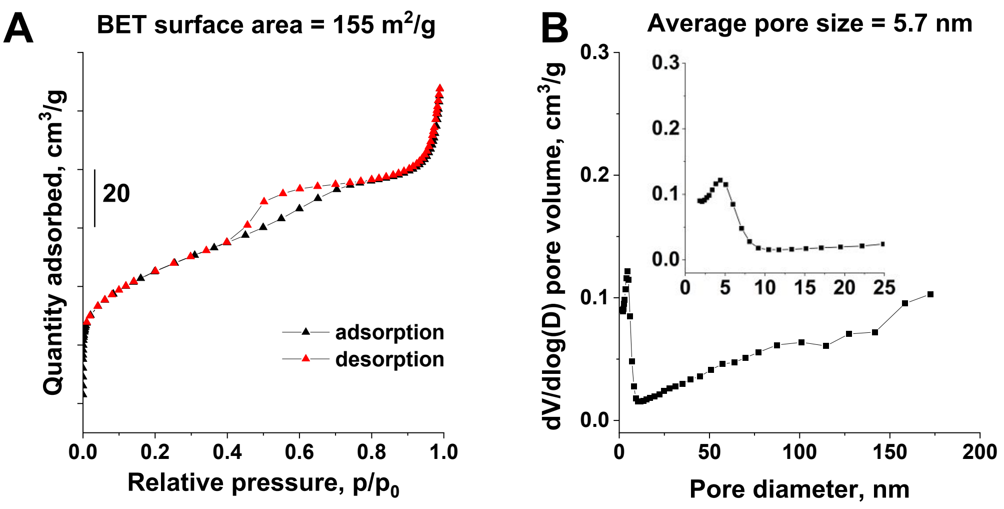


**Figure S1. (A)** Nitrogen adsorption-desorption isotherm and **(B)** pore size distribution estimated for commercial $Nb_2O_5$ CBMM catalyst.

The $N_2$ adsorption-desorption isotherm was obtained at -196°C using a Micromeritics ASAP 2020 Physisorption Analyzer. Before the measurement, the sample was degassed at 120°C for 10 h. The surface area was calculated assuming the BET method and the average pore size was estimated from the adsorption branch using BJH method.

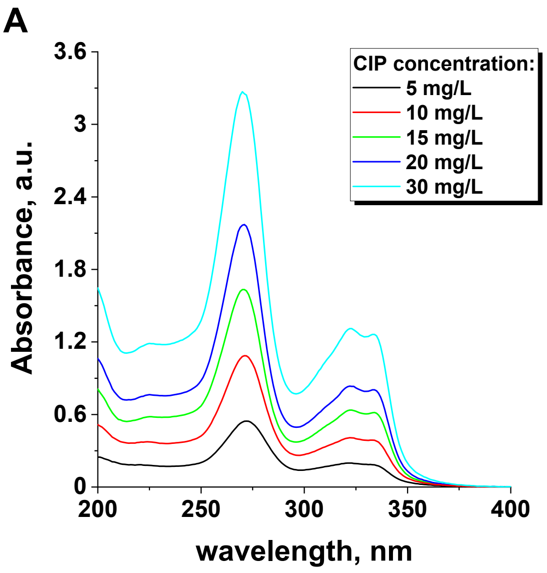


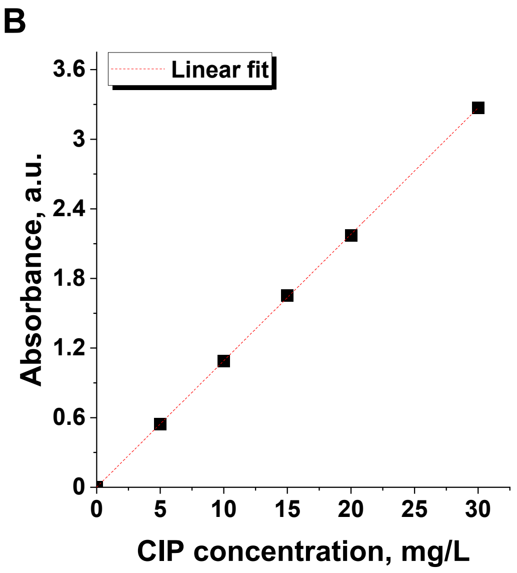


**Figure S2. (A)** UV-vis spectra of ciprofloxacin solution of different concentration. **(B)** Relationship between concentration of the antibiotic and its absorbance at $\lambda_{max} = 270$ nm.

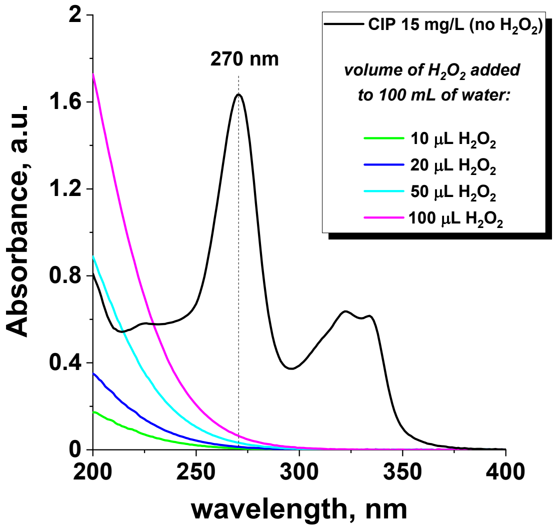


**Figure S3.** Influence of $H_2O_2$ addition on estimation of antibiotic absorbance at $\lambda_{max}$ = 270 nm.

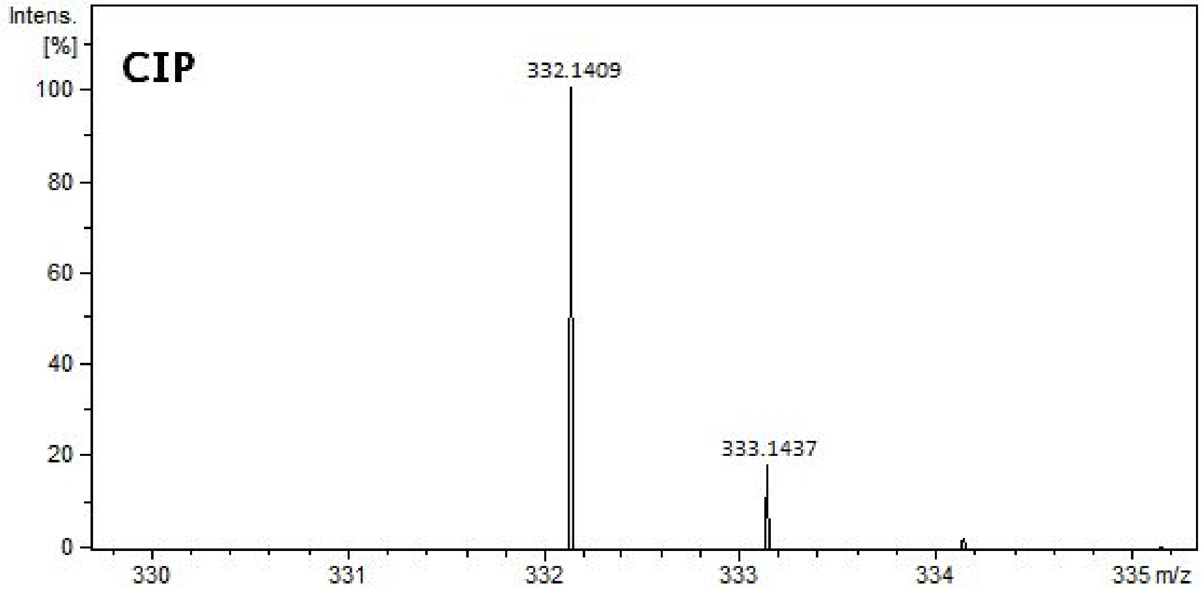


**Figure S4.** The ESI-MS spectrum of the chromatographic peak at 9.4 min, typical of ciprofloxacin.

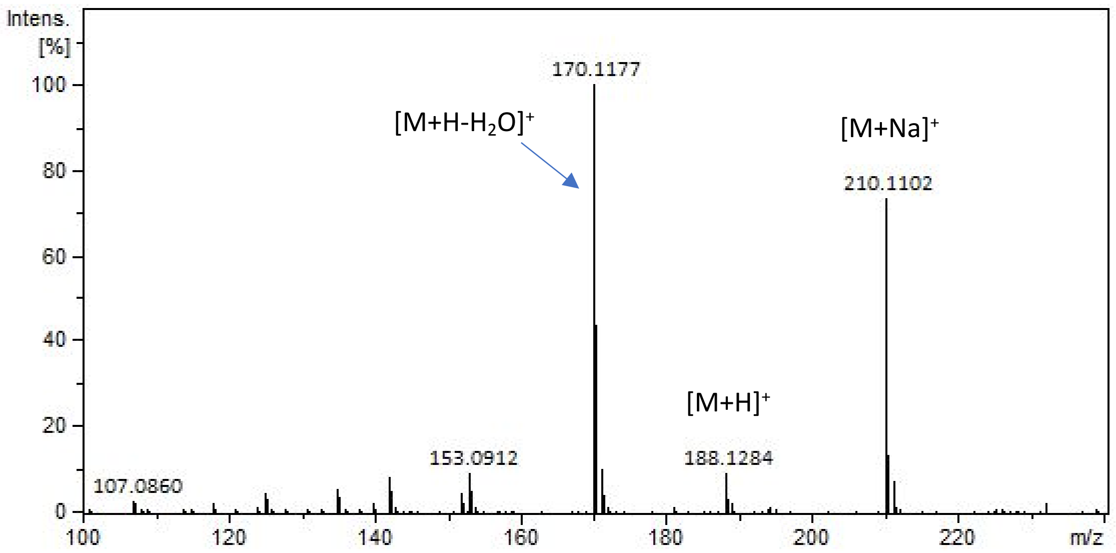


**Figure S5.** The ESI-MS spectrum of the chromatographic peak at 8.5 min.

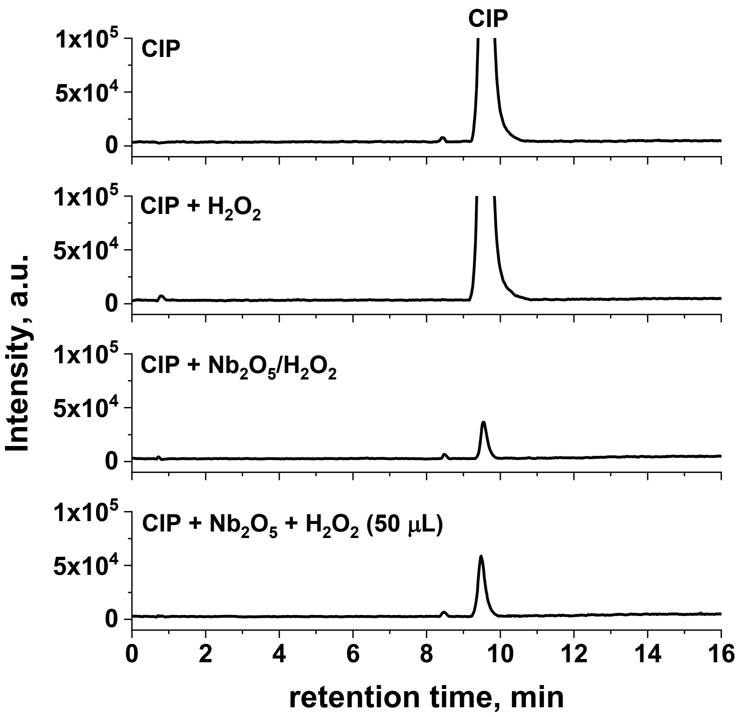


**Figure S6.** LC-MS analyses of CIP and post-reaction solutions shown at higher magnification. Full scale chromatograms are shown in Fig. 3A (see main text of the article).

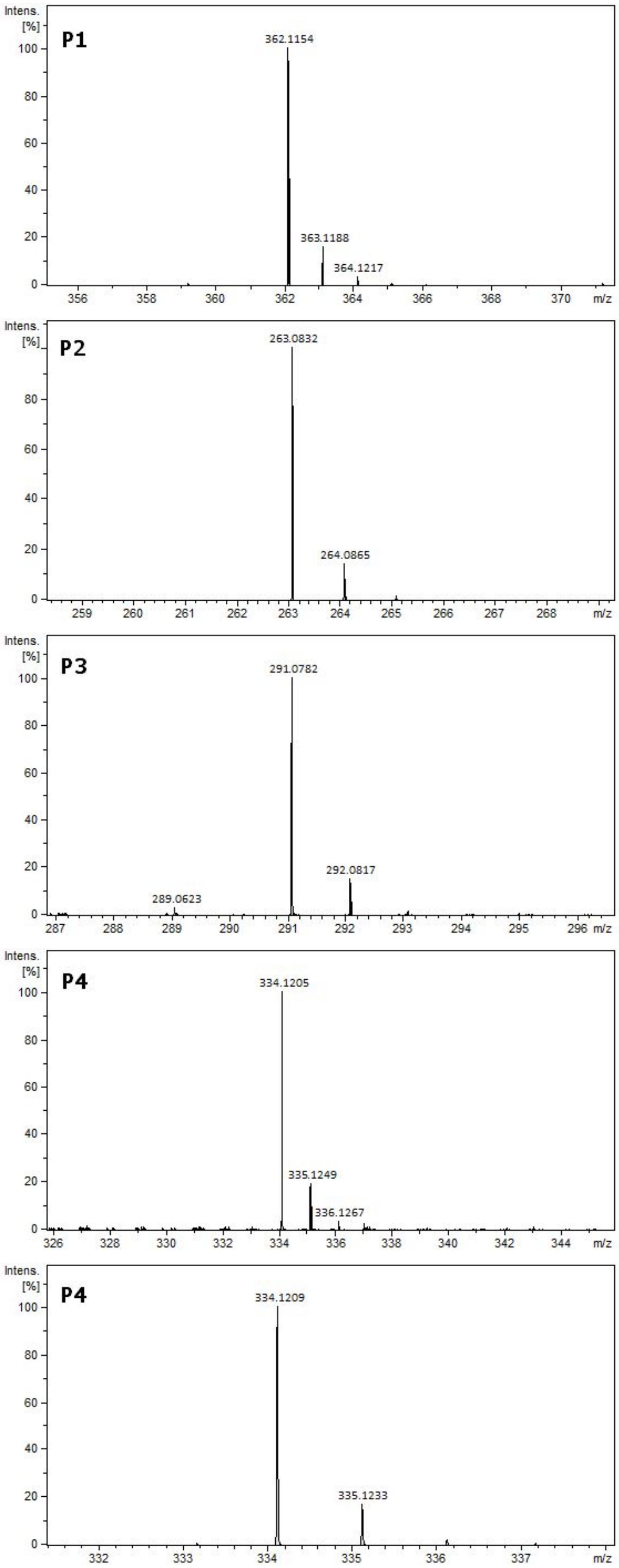


**Figure S7.** The ESI-MS spectra of identified degradation products.

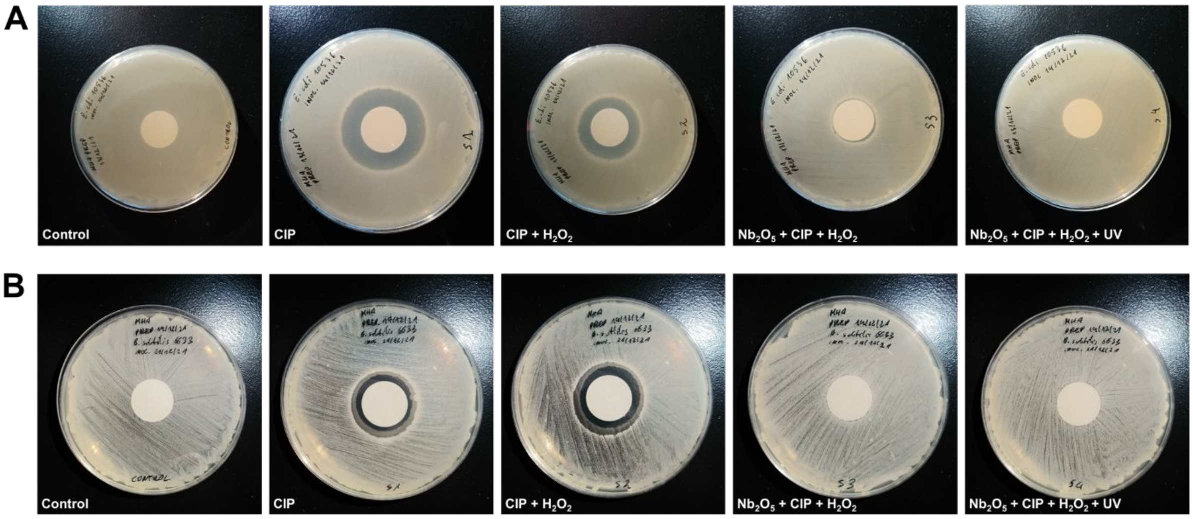


**Figure S8**. Selected images presenting results of antimicrobial tests against **(A)** *E. Coli* and **(B)** *B. Subtilis*.
